# Rapid detection of respiratory organisms with the FilmArray respiratory panel in a large children’s hospital in China

**DOI:** 10.1186/s12879-018-3429-6

**Published:** 2018-10-11

**Authors:** Jin Li, Yue Tao, Mingyu Tang, Bailu Du, Yijun Xia, Xi Mo, Qing Cao

**Affiliations:** 1 Department of Infectious Diseases, Shanghai Children’s Medical Center, Shanghai Jiaotong University School of Medicine, Shanghai, China; 2 The Laboratory of Pediatric Infectious Diseases, Pediatric Translational Medicine Institute, Shanghai Children’s Medical Center, Shanghai Jiaotong University School of Medicine, Shanghai, China; 3 Medical Affairs, Great China | bioMérieux (Shanghai) Company, Limited, Shanghai, China

**Keywords:** Respiratory tract infections, FilmArray respiratory panel, Respiratory organisms, Children

## Abstract

**Background:**

Respiratory tract infections (RTIs) are the most common illness in children, and rapid diagnosis is required for the optimal management of RTIs, especially severe infections.

**Methods:**

Nasopharyngeal swab or sputum specimens were collected from children aged 19 days to 15 years who were admitted to a hospital in Shanghai and diagnosed with RTIs. The specimens were tested with the FilmArray Respiratory Panel, a multiplex PCR assay that detects 16 viruses, *Mycoplasma pneumoniae* (*M. pneumoniae*), *Bordetella pertussis* (*B. pertussis*) and *Chlamydophila pneumoniae* (*C. pneumoniae*).

**Results:**

Among the 775 children studied, 626 (80.8%, 626/775) tested positive for at least one organism, and multiple organisms were detected in 198 (25.5%). Rhinoviruses/enteroviruses (25.5%, 198/775) were detected most often, followed by respiratory syncytial virus (19.5%, 151/775), parainfluenza virus 3 (14.8%, 115/775), influenza A or B (10.9%), adenovirus (10.8%), *M. pneumoniae* (10.6%) and *B. pertussis* (6.3%). The prevalence of organisms differed by age, and most of the viruses were more common in winter. Of the 140 children suspected of having pertussis, 35.0% (49/140) tested positive for *B. pertussis*.

**Conclusions:**

FilmArray RP allows the rapid simultaneous detection of a wide number of respiratory organisms, with limited hands-on time, in Chinese pediatric patients with RTIs.

**Electronic supplementary material:**

The online version of this article (10.1186/s12879-018-3429-6) contains supplementary material, which is available to authorized users.

## Background

Acute respiratory tract infections (RTIs) are the leading causes of outpatient visits and hospitalizations in all age groups, especially during winter and spring. For children under 5 years of age, RTIs are the second leading cause of death [1]. Most acute RTIs in children are caused by respiratory viruses, such as respiratory syncytial virus (RSV), adenovirus (ADV), rhinovirus (RV) and influenza viruses. In addition to viruses, atypical pathogens are major causes of pediatric RTIs. One of the most common atypical pathogens is *Mycoplasma pneumoniae* (*M. pneumoniae*), accounting for 10–40% of hospitalized children with community-acquired pneumonia [2, 3]. In addition to *M. pneumoniae*, the incidence of pertussis in China has significantly increased since 2010. Nevertheless, multiple epidemiological studies have suggested that the incidence of pertussis in China has been significantly underestimated [4, 5]. The early diagnosis of the pathogen is beneficial for the precise selection of medication, which can largely avoid the overuse or even abuse of the antibiotics and improve the clinical care of patients. More importantly, the early diagnosis of contagious pathogens, such as *Bordetella pertussis* (*B. pertussis*) and influenza viruses, can enable early isolation of patients, thus reducing the spread of pathogens.

At present, the routine detection methods for respiratory pathogens in China are mostly based on immunological methods, which include the detection of *M. pneumoniae* and several major viruses, such as RSV, ADV, RV, parainfluenza virus (Para), influenza A virus (FluA) and influenza B virus (FluB). Other respiratory viruses and atypical bacteria, such as *Chlamydophila pneumoniae* (*C. pneumoniae*) and *B. pertussis,* are typically not routinely detected. Given their poor sensitivity and long turn-around time (TAT), immunological methods usually lead to broad-spectrum therapy and have been gradually replaced by molecular-based methods, such as conventional and real-time polymerase chain reaction (PCR), in developed countries [6, 7]. However, most of these molecular tests are technically challenging and require independent spaces, such as pre-PCR and post-PCR rooms, to eliminate the potential risk of cross-contamination, and such requirement limits their applications in China. Therefore, faster, more sensitive and easy-to-use assays for multiplex respiratory pathogen detection are urgently needed.

FilmArray (BioFire Diagnostics, Utah, USA, owned by bioMérieux) is a small, desktop, fully automated multiplex PCR device. The molecular system includes automated nucleic acid extraction, an initial reverse transcription step and multiplex nested PCR, followed by a melting curve analysis [8]. The FilmArray Respiratory Panel (FilmArray RP) is both FDA-approved and CE IVD-marked. The current version of FilmArray RP (v1.7) is able to detect 16 viral and 3 atypical respiratory organisms. The test is performed in a closed system that requires 5 min of hands-on time and 65 min of instrumentation time. Several comparison studies between FilmArray and other tests for respiratory organisms showed comparable results [9–11].

The aim of this study was to evaluate the application of FilmArray RP for the detection of respiratory organisms, and to provide information about the seasonality and prevalence of these organisms in pediatric patients with RTIs in a large children’s hospital in China.

## Methods

### Subjects and specimens

The study population was enrolled according to protocol definitions and inclusion criteria. Patients with respiratory infections, with or without fever (defined as body temperature ≥ 37.5 °C), were included if they had at least one of the following symptoms: (1) cough; (2) nasal obstruction; (3) tachypnoea; (4) nasal flaring; or (5) hypoxia. Patients admitted to the hospital had at least one of the following conditions: (1) unabating high fever; (2) dyspnea, tachypnea or hypoxemia; (3) anorexia or dehydration; (4) radiological confirmation of lung infection; or (5) respiratory infection with underlying diseases, such as congenital heart disease, bronchopulmonary dysplasia, airway malformations, severe malnutrition.

According to the Chinese Center for Disease Control and Prevention (CDC), patients suspected of having pertussis should have a cough for more than 2 weeks and have at least one of the following symptoms: (1) paroxysmal cough; (2) inspiratory whoop; or (3) post-tussive vomiting. In the present study, patients suspected of having pertussis were diagnosed with pertussis when *B. pertussis* was positive by FilmArray RP detection and were otherwise diagnosed with pertussis-like syndrome.

Nasopharyngeal swab (NPS) or sputum specimens were obtained from patients with symptoms of RTIs on the day of hospitalization at Shanghai Children’s Medical Center (SCMC) from December 1, 2016 to November 30, 2017. Demographic data and clinical features, as well as laboratory test and imaging results, were obtained for each enrolled patient. The study was approved by the Institutional Review Board and the Ethics Committee of Shanghai Children’s Medical Center (SCMCIRB-K2017044), and written informed consent was obtained from the parents of each patient.

### FilmArray RP v1.7 testing

The FilmArray RP v1.7 targets 19 organisms, including ADV, influenza A viruses H1, 2009H1, H3 (FluA-H1, FluA-2009H1, FluA-H3) and FluB, parainfluenza virus types 1 to 4 (Para 1–4), coronaviruses 229E, HKU1, OC43, and NL63 (Cov-HKU1, NL63, 229E, OC43), human metapneumovirus (hMPV), RSV, human rhinovirus/enterovirus (Rhino/Entero), *C. pneumoniae*, *M. pneumoniae* and *B. pertussis*. The FilmArray RP assay was performed according to the manufacturer’s instructions. The principle of the assay has been previously described [8, 12]. Each pouch included internal run controls for every step, and results for the assay were only provided by the software if the quality control reactions showed appropriate results.

### Statistical analysis

SPSS software package v21.0 was used for all statistical analyses. Categorical variables were expressed as frequencies and percentages. The chi-square and Fisher’s exact tests were used to compare groups. Continuous variables are expressed as the mean and standard deviation. Student’s *t*-test was used to assess the statistical significance between groups. *p* < 0.05 was considered to be statistically significant.

## Results

### Clinical characteristics of patients

A total of 775 patients diagnosed with upper or lower respiratory tract infections, aged 19 days to 15 years, were enrolled in the present study between December 1, 2016, and November 30, 2017. Congenital heart disease, congenital biliary atresia, malignancy and congenital immunodeficiency were the most frequently observed underlying diseases in these patients and contributed to 50% of the deaths observed in this study. The general characteristics of the patients enrolled are presented in Table 1.

**Table 1:** General characteristics of the patients

Characteristic	Value for patients
Total	775
Age, No. (%)	
< 1 year	338 (43.6)
1–2 years	185 (23.9)
3–5 years	146 (18.9)
6–15 years	106 (13.7)
Sex, No. (%)	
Male	430 (55.5)
Female	345 (44.5)
Clinical diagnosis, No. (%)	
URI	50 (6.5)
LRI	725 (93.5)
Underlying diseases, No. (%)	
None	538 (69.4)
CHD	148 (19.1)
Congenital biliary atresia	25 (3.2)
Malignancy	19 (2.5)
Congenital immunodeficiency	6 (0.8)
Other diseases	39 (5.0)
Prognosis after treat, No. (%)	
Alive	731 (94.3)
Death^b^	12 (1.5)
Unknown^c^	32 (4.1)

^a^Abbreviations: *URI* Upper respiratory tract infection; *LRI* Lower respiratory tract infection; *CHD* Congenital heart disease

^b^Most of the children died from underlying diseases, including malignancy, congenital heart disease, Niemann-Pick Disease, etc.

^c^Including the patients who transfer to other hospitals for treatment or abandon treatment. Upon follow-up by phone, 7 patients showed clinical improvement after transfer to other hospitals, 11 patients died after giving up treatment and discharged from our hospital, and 14 patients cannot be contacted and their prognosis were finally unknown

### Overall detection rate of FilmArray RP v1.7

Among the 775 specimens, 428 (55.2%, 428/775) had a single organism, 198 (25.5%, 198/775) had multiple organisms, and 149 (19.2%, 149/775) had no organism. The overall positive rate of the specimens was 80.8% (626/775). Rhino/Entero was the most prevalent organism (25.5%, 198/775), followed by RSV (19.5%, 151/775) and Para 3 (14.8%, 115/775). The positivity rates of other organisms were as follows: ADV, 10.8% (84/775); *M. pneumoniae*, 10.6% (82/775); *B. pertussis*, 6.3% (49/775); FluA, 6.1% (47/775); FluB, 4.8% (37/775); hMPV, 4.8% (37/775); CoV, 4.3% (33/775); Para 1, 3.2% (25/775); Para 4, 1.3% (10/775); Para 2, 0.4% (3/775); and *C. pneumoniae*, 0.1% (1/775).

### Analysis of the positive rates and prevalence in different age groups

All the patients were grouped by age as follows: infants (age: < 1 year), toddlers (age: 1–2 years), preschoolers (age: 3–5 years) and school-aged children (age: 6–15 years) (Table 2). The highest specimen positivity rate, at 82.2% (278/338), was in the < 1-year age group, followed by 80.5% (149/185), 80.1% (117/146) and 77.4% (82/106) in the 1–2-year, 3–5-year and 6–15-year groups, respectively. There were no significant differences in the positivity rate of the different age groups. In contrast, the prevalence of organisms were different between the different age groups (Table 2). Rhino/Entero, Para 3, RSV and *B. pertussis* showed the highest prevalence in the < 1-year age group, while ADV, hMPV and FluA showed the highest prevalence in the 3–5-year age group. The most prevalent organism in the 6–15-year age group was *M. pneumoniae.* No organism showed a notably high prevalence in the 1–2-year age group. There was only one *C. pneumoniae-*positive patient during the study period, and this patient was in the 3–5-year age group.

**Table 2:** Prevalence of respiratory organisms tested in different age groups

Analyte	< 1 year		1-2 years		3-5 years		6-15 years		χ^2^	*p*
	No. Pos	Prevalence (*n* = 338)	No. Pos	Prevalence (*n* = 185)	No. Pos	Prevalence (*n* = 146)	No. Pos	Prevalence (*n* = 106)		
ADV	17	5.0%	29	15.7%	27	18.5%	11	10.4%	25.157	0.000
CoV total	21	6.2%	7	3.8%	2	1.4%	3	2.8%	6.788	0.079
229E	4	1.2%	1	0.5%	0	0%	2	1.9%	2.750	0.339
HKU1	6	1.8%	1	0.5%	1	0.7%	1	0.9%	1.542	0.696
OC43	11	3.3%	5	2.7%	1	0.7%	0	0%	5.548	0.117
hMPV	9	2.7%	12	6.5%	14	9.6%	1	0.9%	15.751	0.001
Rhino/Entero	101	29.9%	48	25.9%	27	18.5%	22	20.8%	8.453	0.038
FluA total	12	3.6%	13	7.0%	15	10.3%	7	6.6%	8.647	0.034
H3	10	3.0%	11	5.9%	11	7.5%	7	6.6%	5.825	0.120
2009 H1	2	0.6%	2	1.1%	4	2.7%	0	0%	4.550	0.134
FluB	2	0.6%	8	4.3%	15	10.3%	12	11.3%	32.794	0.000
Para total	94	27.8%	36	19.5%	20	13.7%	4	3.8%	34.146	0.000
1	9	2.7%	8	4.3%	8	5.5%	1	0.9%	4.842	0.176
2	0	0%	1	0.5%	1	0.7%	1	0.9%	3.714	0.203
3	81	24.0%	24	13.0%	8	5.5%	2	1.9%	46.976	0.000
4	4	1.2%	3	1.6%	3	2.1%	0	0%	2.095	0.559
RSV	105	31.1%	32	17.3%	11	7.5%	3	2.8%	61.491	0.000
*B. pertussis*	45	13.3%	2	1.1%	1	0.7%	1	0.9%	49.486	0.000
*C. pneumoniae*	0	0%	0	0%	1	0.7%	0	0%	3.750	0.325
*M. pneumoniae*	16	4.7%	16	8.6%	17	11.6%	33	31.1%	60.438	0.000
Total	278	82.2%	149	80.5%	117	80.1%	82	77.4%	1.314	0.726

### Analysis of specimens detected with multiple organisms

Among the 775 specimens, 198 (25.5%, 198/775) were positive for more than one organism. The largest proportion (49.0%, 97/198) of multi-organism-positive specimens had combinations with Rhino/Entero. Rhino/Entero plus Para 3 was the most common combination, making up 10.6% (21/198) of all multi-organism-positive specimens, while the combination of Rhino/Entero plus ADV was the second most common type (6.1%, 12/198), followed by Rhino/Entero plus RSV (5.6%, 11/198). The multi-organism combinations are listed in Additional file 1: Table S1.

### Seasonal prevalence of respiratory organisms from December 1, 2016 to November 30, 2017

**Fig. 1:**
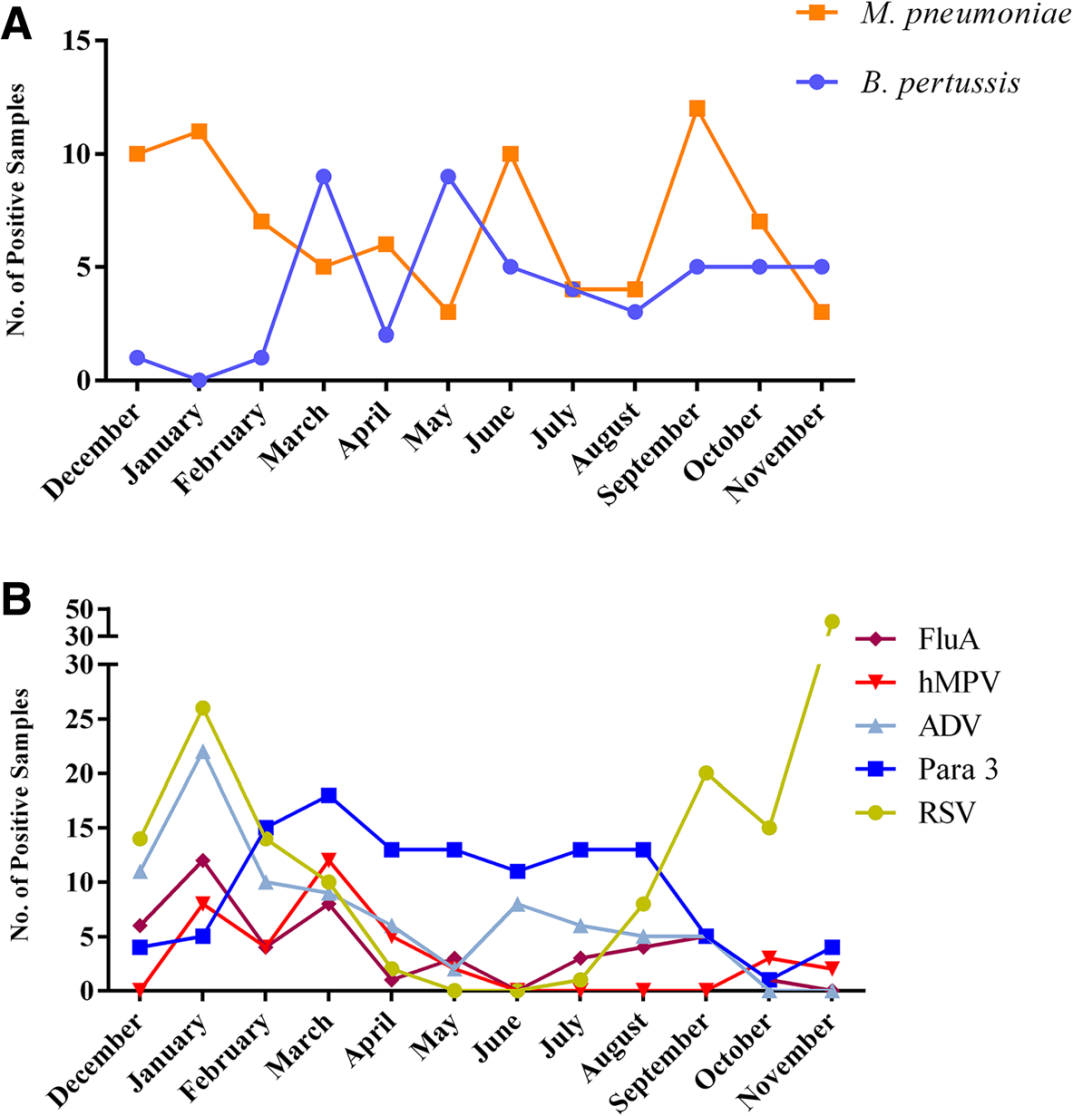
Seasonal distribution of respiratory organisms detected by FilmArray RP. **a** Monthly prevalence of *B. pertussis* and *M. pneumoniae*. **b** Monthly prevalence of FluA, hMPV, ADV, Para 3 and RSV

The number of positive specimens was determined during different months of the year to demonstrate the epidemiology of the respiratory organisms. Regarding the atypical bacteria, *M. pneumoniae* was detected throughout the year, with the highest incidence occurring in September and three minor peaks in December, January and June (Fig. 1a). The highest incidences of *B. pertussis* were observed in March and May. Only one case of *C. pneumoniae* was detected in July.

The seasonal prevalence of viruses with high detection rates were as follows. Both FluA and hMPV had two peaks that occurred in January and March, and ADV showed a peak in January (Fig. 1b). The prevalence of Para 3 remained high from February–August. The peaks in RSV cases occurred in both the fall and the winter months. The number of Rhino/Entero cases was relatively high throughout the year regardless of season.

### The detection rates for the NPS and sputum samples

Among the 775 patients in our study, NPS samples were collected from 662 (85.4%). Among the other 113 (14.6%) patients, 88 used ventilators, 13 were hypoxic, and 12 were cyanotic after spasmodic coughing or crying, preventing NPS samples from being obtained from these patients; instead, sputum samples were collected and sent for FilmArray RP detection. The positivity rates of the NPS and sputum samples were 83.5% (553/662) and 64.6% (73/113), respectively. Detailed organism information on the two sample types collected from the different age groups is provided in Additional file 2: Tables S2 and S3.

### Respiratory organisms detected in patients with suspected pertussis

**Fig. 2:**
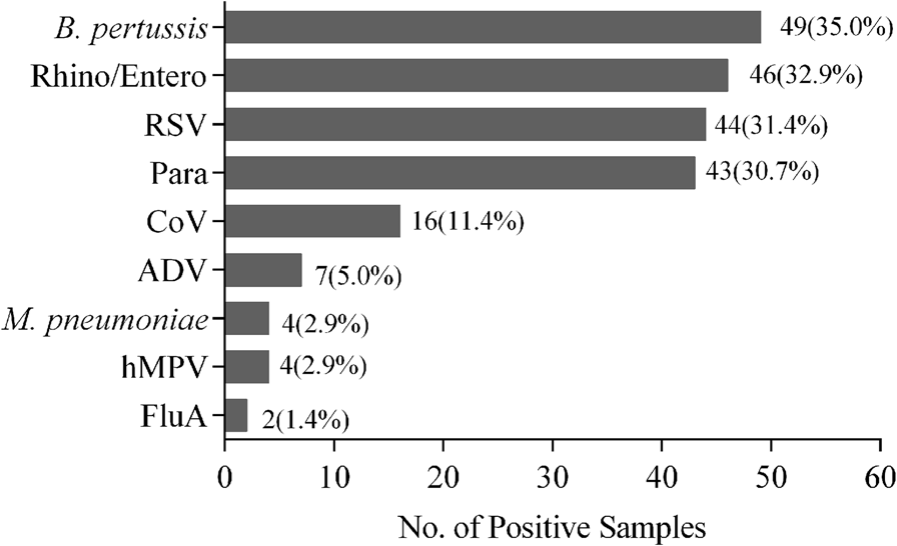
Detection of respiratory organisms in 140 children suspected with pertussis. 35% (49/140) of the children were tested positive for *B. pertussis* with FilmArray RP

According to the government policy, all cases of positive pertussis have to be reported to CDC. Because no routine examination could distinguish the patients with pertussis from those with pertussis-like syndrome, we paid special attention to the 140 patients who were clinically diagnosed with suspected pertussis in the present study. Among these patients, 95.0% (133/140) were positive for at least one organism by FilmArray RP, with 50.0% (70/140) and 45.0% (63/140) having single and multiple organisms detected, respectively. Detailed information on the organisms detected is presented in Fig. 2. 49 in the 140 patients (35%) were detected pertussis positive, among whom 42 (85.7%) were under 6 months, and 25 (71.4%) were co-detected with at least one virus.

## Discussion

In our study, 775 specimens were collected from pediatric patients with RTIs over a period of one year and analyzed with FilmArray RP v1.7. The overall results yielded a positivity rate of 80.8%, with multiple organisms detected in 25.5% of specimens, which is in accordance with Litwin and Piralla’s reports [13, 14]. As in other studies, a notable variation in the pathogen prevalence with season and age was observed. Most viruses had their highest positivity rates in winter, except that Para 3 positivity rate was well distributed through the spring and summer, and the epidemiologic peaks for hMPV occurred 1 to 2 months later than those for RSV [15, 16]. The majority of respiratory viruses were observed in children younger than 5 years old. Notably, RSV was the most prevalent virus in the < 1-year age group, and the prevalence decreased with age; while the incidence of *M. pneumoniae* increased with age [17]*.*

Multiple respiratory organisms were detected in 25.5% of the specimens in our study, the largest proportion of which included Rhino/Entero. Other studies in adults reported lower multi-pathogen detection rates of approximately 8.7–15.9% [14, 18–20], suggesting that pediatric patients with RTIs are more likely to be infected by multiple pathogens than adults. However, the clinical significance, including disease severity and hospitalization time, of multi-pathogen infection, especially Rhino/Entero combination infections, is not clear. A previous report indicated that dual-positive results with RSV and Rhino/Entero specimens might be due to viral shedding from a previous Rhino/Entero infection [21]. Nokso-Koivisto et al. also found that rhinovirus was the most prevalent virus in asymptomatic carriers [22].

The most unexpected result in our study is the high detection rate of *B. pertussis,* with an overall detection rate of 6.32% in the group of 775 patients, further demonstrating the value of FilmArray RP in clinical application. At present, the diagnosis of pertussis in China is based on culture and serology results. However, both the CDC and World Health Organization (WHO) use positive PCR results as the criteria for diagnosis, suggesting that FilmArray RP testing, in addition to culture, can be considered for patients with suspected pertussis in order to better monitor disease outbreaks. Additionally, the early diagnosis of patients with *B. pertussis*, which is typically difficult to distinguish from pertussis-like syndrome, can also help to reduce unnecessary macrolide treatment. The limitation of the panel is the lack of *B. parapertussis,* which contributes to more than 5% of pertussis cases [23]. However, it has been added to the second-generation panel, FilmArray RP2 v1.1 [24], and the prevalence of *B. parapertussis* in our patients is currently under investigation.

As stated in the manufacturer’s instructions, “FilmArray Respiratory Panel (RP) is a multiplexed nucleic acid test intended for use with FilmArray systems for the simultaneous qualitative detection and identification of multiple respiratory viral and bacterial nucleic acids in nasopharyngeal swabs (NPS) obtained from individuals suspected of respiratory tract infections”. Therefore, NPS samples are recommended for FilmArray RP, but there are also studies demonstrating a comparable or even higher detection rate in sputum [25, 26]. However, the detection rate in sputum in our study was lower than that in NPS samples. This might partially be attributed to the fact that most of the sputum samples (86.7%, 98/113) were from ICU patients, and the sputum-providing patients showed a higher positivity rate in their sputum culture than the NPS-providing patients (33.6% vs 20.5%). In addition to sputum, bronchoalveolar lavage fluid (BALF) is a common type of respiratory sample, and Azadah et al. showed that detection in BALF by FilmArray RP can provide new and useful microbiological information within 7 days after a negative NPS result is obtained [27]. Therefore, the choice of the most appropriate sample type and time-point for each patient, particularly in specific clinical contexts, such as undergoing fiberoptic bronchoscopy or ventilator use, may require further investigation.

As with other molecular methods, distinguishing whether the microbes detected in the FilmArray analysis, especially those that are also detected in asymptomatic children, such as human rhinovirus, are causative pathogens or colonizers is not feasible [28–30]. Therefore, the clinicians should take caution when judging pathogens because the results are sometimes “false positive”. On the other hand, despite the high detection rate of FilmArray RP, a negative result does not mean the patient is not infected; moreover, a positive result does not mean there is no other co-infecting agent, especially in critically ill patients, in whom a bacterial co-infection often occurs. For this “false-negative” limitation, BioFire has a new pneumonia panel that also targets BALF/sputum and covers 9 common viruses, as well as 15 bacteria, including *Klebsiella pneumonia*, *Haemophilus influenza*, *Streptococcus pneumonia* and *Staphylococcus aureus*. Nevertheless, the FilmArray panel only aims to rapidly provide results for potential pathogens as a reference. A more appropriate method is to comprehensively consider the results from other examinations, such as routine blood testing, C-reactive protein (CRP), procalcitonin (PCT), the erythrocyte sedimentation rate (ESR), culture and radiography, as well as the patients’ symptoms, including body temperature, breathing, blood oxygen, heart rate, and mental condition.

Our study also has several limitations. First, our study was performed in a single center and may not be representative of the entire Chinese pediatric population. Second, we did not have data from a more appropriate assay to evaluate the specificity of FilmArray RP. Additionally, we do not provide detailed information on the effects of FilmArray RP on the use of antibiotics, clinical outcomes and health economics, which require further investigation.

## Conclusion

In conclusion, the FilmArray RP assay significantly expands our ability to diagnose multiple respiratory infections caused by viruses and atypical bacteria. The array can detect 19 respiratory organisms simultaneously, with a high detection rate, in 65 min. Our study provided the age groups and seasonal distributions of different organisms for pediatric RTI patients. This study also provides new insights into the current status of pertussis infection in China. Whether FilmArray RP can enhance clinical decision-making and limit the unnecessary use of antibiotics in China as in other countries still requires further investigation.

## Additional files

Additional file 1: **Table S1.** Combinations of multiple organisms detected with FilmArray RP. (DOC 99 kb)

Additional file 2: **Table S2.** Overall detection rates for the nasopharyngeal swab and sputum samples from the different age groups. **Table S3.** The respiratory organisms in the nasopharyngeal swab and sputum samples from the different age groups detected with FilmArray RP. (DOC 72 kb)

## Abbreviations

ADV: Adenovirus
*B. pertussis*: *Bordetella pertussis*
*C. pneumoniae*: *Chlamydophila pneumoniae*
CAP: Community-acquired pneumonia
CoV: Coronaviruses
FilmArray RP: FilmArray Respiratory Panel
FluA: Influenza A viruses
FluB: Influenza B viruses
hMPV: Human metapneumovirus
*M. pneumoniae*: *Mycoplasma pneumoniae*
NPS: Nasopharyngeal swab
Para: Parainfluenza virus
PCR: Polymerase chain reaction
Rhino/Entero: Human rhinovirus/enterovirus
RITs: Respiratory tract infections
RSV: Respiratory syncytial virus
SCMC: Shanghai Children’s Medical Center
