# Supplementary material for: Rapid detection of respiratory organisms with the FilmArray respiratory panel in a large children’s hospital in China
Source: BMC Infect Dis. 2018 Oct 11;18:510. doi: 10.1186/s12879-018-3429-6 (PMC6180626; doi:10.1186/s12879-018-3429-6)
Supplement: Supplementary file 1 — Table S1. Combinations of multiple organisms detected with FilmArray RP. (DOC 99 kb) [file 12879_2018_3429_MOESM1_ESM.doc]

Table S1 Combinations of multiple organisms detected with FilmArray RP.

| **Analyte 1** | **Analyte 2** | **Analyte 3** | **Analyte 4** | **No. of multi-analyte positive samples** |
| --- | --- | --- | --- | --- |
| Rhino/Entero | Para 3 |  |  | 21 |
|  | ADV |  |  | 12 |
|  | RSV |  |  | 11 |
|  | *B. pertussis* |  |  | 8 |
|  | *M. pneumoniae* |  |  | 7 |
|  | *B. pertussis* | Para 3 |  | 5 |
|  | Para 3 | ADV |  | 3 |
|  | FluB |  |  | 2 |
|  | Para 1 |  |  | 2 |
|  | hMPV |  |  | 2 |
|  | RSV | ADV |  | 2 |
|  | *B. pertussis* | RSV |  | 2 |
|  | RSV | Para 3 |  | 2 |
|  | *M. pneumoniae* | Para 3 |  | 2 |
|  | FluA |  |  | 1 |
|  | CoV-HKU1 |  |  | 1 |
|  | Para 4 |  |  | 1 |
|  | hMPV | Para 3 |  | 1 |
|  | RSV | hMPV |  | 1 |
|  | *M. pneumoniae* | RSV |  | 1 |
|  | ADV | hMPV |  | 1 |
|  | ADV | Para 4 |  | 1 |
|  | *B. pertussis* | ADV |  | 1 |
|  | *B. pertussis* | CoV-HKU1 |  | 1 |
|  | hMPV | *B. pertussis* |  | 1 |
|  | hMPV | FluA |  | 1 |
|  | CoV-HKU1 | Para 1 |  | 1 |
|  | RSV | CoV-229E |  | 1 |
|  | *M. pneumoniae* | FluA | ADV | 1 |
|  | Para 3 | CoV-HKU1 | *B. pertussis* | 1 |
| Para 3 | RSV |  |  | 9 |
|  | ADV |  |  | 8 |
|  | 1. *pneumoniae* |  |  | 4 |
|  | 1. *pertussis* |  |  | 3 |
|  | FluB |  |  | 2 |
|  | CoV-229E |  |  | 1 |
|  | CoV-OC43 |  |  | 1 |
|  | CoV-HKU1 |  |  | 1 |
|  | hMPV |  |  | 1 |
|  | Para 1 |  |  | 1 |
|  | FluA |  |  | 1 |
|  | Para 3 | ADV |  | 1 |
|  | Para 3 | *B. pertussis* |  | 1 |
|  | FluA | ADV |  | 1 |
|  | CoV-OC43 | *B. pertussis* |  | 1 |
|  | CoV-OC43 | CoV-HKU1 |  | 1 |
|  | Para 4 | *B. pertussis* |  | 1 |
|  | CoV-OC43 | CoV-229E | *B. pertussis* | 1 |
| RSV | *B. pertussis* |  |  | 5 |
|  | *M. pneumoniae* |  |  | 4 |
|  | CoV-OC43 |  |  | 3 |
|  | Para 4 |  |  | 2 |
|  | *M. pneumoniae* | ADV |  | 2 |
|  | FluB |  |  | 1 |
|  | Para 1 |  |  | 1 |
|  | Para 2 |  |  | 1 |
|  | FluA |  |  | 1 |
|  | ADV |  |  | 1 |
|  | CoV-229E |  |  | 1 |
|  | *M. pneumoniae* | Para 1 |  | 1 |
|  | FluA | hMPV |  | 1 |
|  | Para 1 | FluB | ADV | 1 |
|  | Para 4 | FluA | ADV | 1 |
| ADV | *M. pneumoniae* |  |  | 7 |
|  | FluA |  |  | 4 |
|  | CoV-OC43 |  |  | 3 |
|  | hMPV |  |  | 3 |
|  | Para 1 |  |  | 1 |
|  | FluB |  |  | 1 |
|  | *B. pertussis* |  |  | 1 |
| *M. pneumoniae* | CoV-OC43 |  |  | 2 |
|  | Para 1 |  |  | 1 |
|  | Para 4 |  |  | 1 |
|  | FluB |  |  | 1 |
|  | FluA |  |  | 1 |
|  | CoV-229E |  |  | 1 |
| FluA | Para 4 |  |  | 1 |
|  | FluB |  |  | 1 |
|  | CoV-229E |  |  | 1 |
| hMPV | Para 4 |  |  | 1 |
|  | FluB |  |  | 1 |
|  | CoV-HKU1 |  |  | 1 |
| CoV-OC43 | *B. pertussis* |  |  | 2 |
|  | CoV-229E | *B. pertussis* |  | 1 |
