# Supplementary material for: Rapid detection of respiratory organisms with the FilmArray respiratory panel in a large children’s hospital in China
Source: BMC Infect Dis. 2018 Oct 11;18:510. doi: 10.1186/s12879-018-3429-6 (PMC6180626; doi:10.1186/s12879-018-3429-6)
Supplement: Supplementary file 2 — Table S2. Overall detection rates for the nasopharyngeal swab and sputum samples from the different age groups. Table S3. The respiratory organisms in the nasopharyngeal swab and sputum samples from the different age groups detected with FilmArray RP. (DOC 72 kb) [file 12879_2018_3429_MOESM2_ESM.doc]

Table S2 Overall detection rates for the nasopharyngeal swab and sputum samples from the different age groups.

| **Age group** | **Nasopharyngeal swab samples** | | **Sputum samples** | | **χ^2^** | ***p*-value** |
| --- | --- | --- | --- | --- | --- | --- |
|  | No. of samples | No. (%) of positive samples | No. of samples | No. (%) of positive samples |  |  |
| <1 year | 265 | 230 (86.8%) | 73 | 48 (65.8%) | 17.352 | 0.000 |
| 1-2 years | 162 | 135 (83.3%) | 23 | 14 (60.9%) | 5.131^a^ | 0.024 |
| 3-5 years | 139 | 113 (81.3%) | 7 | 4 (57.1%) | 1.161^a^ | 0.281 |
| 6-15 years | 96 | 75 (78.1%) | 10 | 7 (70.0%) | 0.035^a^ | 0.851 |
| **Total** | **662** | **553 (83.5%)** | **113** | **73 (64.6%)** | **22.280** | **0.000** |

^a^ Correction for continuity

Table S3 The respiratory organisms in the nasopharyngeal swab and sputum samples from the different age groups detected with FilmArray RP.

| **Analyte** | **<1 year** | | | | **1-2 years** | | | | **3-5 years** | | | | **6-15 years** | | | |
| --- | --- | --- | --- | --- | --- | --- | --- | --- | --- | --- | --- | --- | --- | --- | --- | --- |
|  | NPS (n=265) | | Sputum (n=73) | | NPS (n=162) | | Sputum (n=23) | | NPS (n=139) | | Sputum (n=7) | | NPS (n=96) | | Sputum (n=10) | |
|  | No. | Pos.% | No. | Pos.% | No. | Pos.% | No. | Pos.% | No. | Pos.% | No. | Pos.% | No. | Pos.% | No. | Pos.% |
| ADV | 15 | 5.7% | 2 | 2.7% | 26 | 16.0% | 3 | 13.0% | 26 | 18.7% | 1 | 14.3% | 11 | 11.5% | 0 | 0 |
| CoV total | 20 | 7.5% | 1 | 1.4% | 7 | 4.3% | 0 | 0 | 2 | 1.4% | 0 | 0 | 2 | 2.1% | 1 | 10.0% |
| 229E | 4 | 1.5% | 0 | 0 | 1 | 0.6% | 0 | 0 | 0 | 0 | 0 | 0 | 2 | 2.1% | 0 | 0 |
| HKU1 | 6 | 2.3% | 0 | 0 | 1 | 0.6% | 0 | 0 | 1 | 0.7% | 0 | 0 | 0 | 0 | 1 | 10.1% |
| OC43 | 10 | 3.8% | 1 | 1.4% | 5 | 3.1% | 0 | 0 | 1 | 0.7% | 0 | 0 | 0 | 0 | 0 | 0 |
| hMPV | 6 | 2.3% | 3 | 4.1% | 11 | 6.8% | 1 | 4.3% | 13 | 9.4% | 1 | 14.3% | 1 | 1.0% | 0 | 0 |
| Rhino/Entero | 89 | 33.6% | 12 | 16.4% | 41 | 25.3% | 7 | 30.4% | 25 | 18.0% | 2 | 28.6% | 18 | 18.8% | 4 | 40.0% |
| FluA total | 11 | 4.2% | 1 | 1.4% | 11 | 6.8% | 2 | 8.7% | 15 | 10.8% | 0 | 0 | 6 | 6.3% | 1 | 10.0% |
| H3 | 10 | 3,8% | 0 | 0 | 10 | 6.2% | 1 | 4.3% | 11 | 7.9% | 0 | 0 | 6 | 6.3% | 1 | 10.0% |
| 2009H1 | 1 | 0.4% | 1 | 1.4% | 1 | 0.6% | 1 | 4.3% | 4 | 2.9% | 0 | 0 | 0 | 0 | 0 | 0 |
| FluB | 1 | 0.4% | 1 | 1.4% | 7 | 4.3% | 1 | 4.3% | 15 | 10.8% | 0 | 0 | 12 | 12.5% | 0 | 0 |
| Para total | 75 | 28.3% | 19 | 26.0% | 31 | 19.1% | 5 | 21.7% | 20 | 14.4% | 0 | 0 | 3 | 3.1% | 1 | 10.0% |
| 1 | 6 | 2.3% | 3 | 4.1% | 8 | 4.9% | 0 | 0 | 8 | 5.8% | 0 | 0 | 1 | 1.0% | 0 | 0 |
| 2 | 0 | 0 | 0 | 0 | 1 | 0.6% | 0 | 0 | 1 | 0.7% | 0 | 0 | 1 | 1.0% | 0 | 0 |
| 3 | 65 | 24.5% | 16 | 21.9% | 20 | 12.3% | 4 | 17.4% | 8 | 5.8% | 0 | 0 | 1 | 1.0% | 1 | 10.0% |
| 4 | 4 | 1.5% | 0 | 0 | 2 | 1.2% | 1 | 4.3% | 3 | 2.2% | 0 | 0 | 0 | 0 | 0 | 0 |
| RSV | 87 | 32.8% | 18 | 24.7% | 31 | 19.1% | 1 | 4.3% | 9 | 6.5% | 2 | 28.6% | 3 | 3.1% | 0 | 0 |
| 1. *pertussis* | 41 | 15.5% | 4 | 5.5% | 2 | 1.2% | 0 | 0 | 1 | 0.7% | 0 | 0 | 1 | 1.0% | 0 | 0 |
| 1. *pneumoniae* | 0 | 0 | 0 | 0 | 0 | 0 | 0 | 0 | 1 | 0.7% | 0 | 0 | 0 | 0 | 0 | 0 |
| *M. pneumoniae* | 14 | 5.3% | 2 | 2.7% | 14 | 8.6% | 2 | 8.7% | 17 | 12.2% | 0 | 0 | 31 | 32.3% | 2 | 20.0% |

Abbreviations: NPS, nasopharyngeal swab; Pos.%, positive rate
